# *Wolbachia* Can Enhance *Plasmodium* Infection in Mosquitoes: Implications for Malaria Control?

**DOI:** 10.1371/journal.ppat.1004182

**Published:** 2014-09-04

**Authors:** Grant L. Hughes, Ana Rivero, Jason L. Rasgon

**Affiliations:** 1 The Huck Institutes of The Life Sciences, The Center for Infectious Disease Dynamics and the Department of Entomology, Pennsylvania State University, State College, Pennsylvania, United States of America; 2 Maladies Infectieuses et Vecteurs: Écologie, Génétique, Évolution et Contrôle, MIVEGEC (UMR CNRS-UM1-UM2 5290, IRD 224), Montpellier, France; The Fox Chase Cancer Center, United States of America

The symbiotic bacterium *Wolbachia* is an attractive agent for vector-borne pathogen control. It has long been studied for its ability to manipulate host reproduction and spread into arthropod populations [1]. These properties, coupled with the recently identified ability to inhibit diverse pathogens [2]–[6], open avenues for its use in controlling vector-borne disease. Numerous *Wolbachia*-based control strategies are being investigated (reviewed in [7]–[9]), with some studies having progressed to field trials [10], [11]. However, a worrying trend is emerging whereby *Wolbachia* infections have been demonstrated to enhance rather than suppress pathogens in some systems [12]–[18]. *Plasmodium* parasites, which are the causal agent of malaria, seem particularly prone to *Wolbachia*-mediated pathogen enhancement [13]–[16].

*Wolbachia*-based strategies have been proposed to control malaria [19]. *Anopheles* mosquitoes (the vectors of human malaria parasites) are not naturally infected by *Wolbachia* [20], [21], but artificial transfer of this bacterium between species can be accomplished in the laboratory (reviewed in [22]). Pathogen interference phenotypes appear to be most prominent when *Wolbachia* is transferred into a novel host [16], [23]. Given that *Anopheles* are for the most part naturally uninfected by *Wolbachia* (but see [24]), they can be considered an open niche for infection and a prime mosquito genus for *Wolbachia*-based control strategies. However, the main impediment for developing a control strategy is the difficulty in creating a stable artificial infection in *Anopheles* [19]. While examining *Plasmodium* interference in a stably infected host is the gold standard, a more convenient system is to transiently infect mosquitoes by intrathoracic microinjection. Using this system, the infection persists during the lifetime of the transinfected individual but is not transmitted to its offspring. Transient infection allows the rapid assessment of *Wolbachia*-host interactions without the need for generating stable artificial infections [5]. It is uncertain how representative transient infections are of stable inherited associations; however, similarities in tissues tropism and fitness costs incurred upon the host between stable and transiently infected *Anopheles* mosquitoes are evident [5], [14], [25]. Furthermore, both types of infection have been shown to inhibit the human malaria parasite *Plasmodium falciparum* [5], [25]. However, studies using transient infection models have found that *Wolbachia* can enhance certain *Plasmodium* species [13], [14].

The *Plasmodium* interference phenotype is therefore not universal, but context dependent. While *P. falciparum* is suppressed by the *w*AlbB strain of *Wolbachia* from *Aedes albopictus* [5], [25], transient infections have shown the opposite effect on rodent malaria parasites. *Anopheles gambiae* transiently infected with *w*AlbB exhibited enhanced *P. berghei* development at the oocyst stage [14]. Similarly, *w*AlbB increased the number of *P. yoelii* oocysts in *An. stephensi*, although the phenotype was modulated by temperature [13]. At a temperature optimal for parasite development, *Wolbachia* increased parasite intensity compared to uninfected controls, but at warmer temperatures, *Wolbachia* inhibited *Plasmodium* development [13].

**Figure 1:**
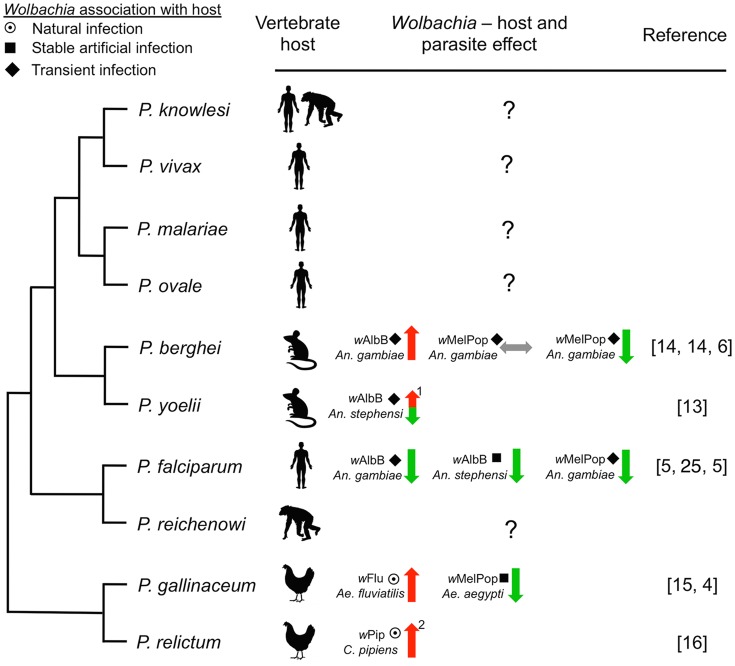
Representative phylogenetic dendrogram of *Plasmodium* parasites, their vertebrate hosts, and the influence of *Wolbachia* infection on parasite development within the mosquito vector. The protective effect of *Wolbachia* is variable and dependent on the *Wolbachia* strain and the insect host background, suggesting that complex tripartite interactions influence the effect on *Plasmodium*. The type of association between *Wolbachia* with the vector may also influence *Plasmodium*. Only one human malaria parasite (*P. falciparum*) has been assessed, while the effect of *Wolbachia* infection on the other four human parasites is unknown. Arrows indicate suppression (green), enhancement (red), or no effect (grey) of *Plasmodium*. The type of association within the host is depicted by symbols (target: natural infection, square: stable artificial infection, diamond: transient artificial infection). Numbers indicate: (1) the phenotype is temperature sensitive, (2) *Wolbachia* infection also increases insect life span [31], which has implications for pathogen transmission. Phylogeny was reconstructed based on work from Carlton et al. [26] and Martinsen et al. [27].

While *P. falciparum* is a major parasite in sub-Saharan Africa, four other parasites also cause human malaria worldwide: *P. malariae*, *P. ovale*, *P. knowlesi,* and *P. vivax* (the etiological agent of the most prevalent form of relapsing malaria). To our knowledge, the effect of *Wolbachia* on these other human *Plasmodium* parasites is unknown. The question is relevant for two reasons. First, the precedent that a particular *Wolbachia* strain can inhibit one parasite yet enhance another has already been documented [5], [14], indicating that effects on parasites can be species-specific. Troublingly, *P. malariae*, *P. ovale*, *P. knowlesi*, and *P. vivax* are phylogenetically more closely related to rodent malaria parasites, which are enhanced by *Wolbachia* infections [13], [14], than they are to *P. falciparum* (Figure 1) [26], [27]. Second, many human *Plasmodium* parasites occur in sympatry and are transmitted by the same vectors. A case in point is *P. falciparum* and *P. vivax*, both of which occur in sympatry over large stretches of the Asian continent where they are both transmitted by *An. stephensi* [28], [29]. Any potential control strategy devised in regions where more than one parasite species occurs needs to thoroughly investigate the effect of *Wolbachia* on all parasite species transmitted by the vector, as well as other pathogens such as filarial worms or arboviruses (both as single infections and in the context of coinfections) to ensure that *Wolbachia*-infected mosquitoes do not inadvertently enhance transmission of secondary pathogens.

While difficult, forecasting the long-term evolutionary response in this tripartite relationship between *Wolbachia*, *Plasmodium,* and *Anopheles* is very important. Natural *Wolbachia*–mosquito associations in which the symbiont and the host have tightly coevolved exist and may provide powerful models for studying the long-term evolutionary effects of *Wolbachia* infections. The evidence currently available suggests that natural *Wolbachia* infections can also enhance malaria parasite development within the mosquito. *Aedes fluviatilis* naturally infected with the *w*Flu *Wolbachia* strain had a significantly higher number of *P. gallinaceum* oocysts compared to an *Ae. fluviatilis* line which had been cleared of the *Wolbachia* infection [15]. *Ae. fluviatilis* is not, however, a natural vector of *P. gallinaceum*, and it is well known that the outcome of experiments using such laboratory models can differ significantly from those of natural mosquito–*Plasmodium* combinations (e.g., Boete [30]). Recent studies carried out in *Culex pipiens* mosquitoes, which are naturally infected with the *w*Pip *Wolbachia* strain and transmit the avian malaria parasite *P. relictum*, have also demonstrated *Plasmodium* enhancement. In this natural system, *Wolbachia* protects the mosquito host against the detrimental fitness effects incurred by *Plasmodium* infection [31] and increases the susceptibility of *C. pipiens* to *P. relictum*, with *w*Pip-infected mosquitoes having a higher prevalence of *Plasmodium* sporozoites in the salivary glands [16]. These studies show that the *Plasmodium*-inhibiting properties of *Wolbachia* are far from universal; certain mosquito–*Wolbachia*–*Plasmodium* combinations and experimental conditions transform *Wolbachia*-infected mosquitoes into better vectors of malaria parasites. This is worrisome for the general implementation of *Wolbachia*-based control strategies.

Given that *Wolbachia*-based control strategies will use stable transinfected mosquitoes, the key question is whether stable and natural infections will behave in the same way. The stable transfer of *Wolbachia* into the host likely alters many aspects of host homeostasis, as evidenced by the novel phenotypes induced by infection [32]–[34], and as such, these associations likely differ from natural associations where *Wolbachia* and its host have coevolved. Another question is whether stable artificial infections will evolve over time. Theory and empirical studies show that these maternally transmitted bacteria will tend to evolve towards mutualistic associations with their host [35]–[38]. However, the evolutionary outcomes of pathogen interference or enhancement are harder to predict. A more complete mechanistic understanding of how *Wolbachia* infection modulates *Plasmodium* parasites is critical to address these important evolutionary questions and to evaluate if they are likely to occur in timescales relevant for disease control.

To date, two stable artificial *Wolbachia* transinfections have been assessed for their effect on *Plasmodium*. First, an *Aedes aegypti* line infected with *w*MelPop had inhibited *P. gallinaceum* infection [4]; *Ae. aegypti* is not, however, the natural vector of this parasite. Second, and more recently, the *w*AlbB strain was stably transferred into *An. stephensi*, one of the main vectors of human malaria in Asia [25]. This groundbreaking work demonstrated that stable artificial infections in epidemiologically relevant malaria vectors are feasible, and that *P. falciparum* can be inhibited by *Wolbachia* within its natural vector. If the severe fitness effects induced by *Wolbachia* in *Anopheles* can be overcome [25], then this approach is highly promising.

The work by Bian and colleagues [25] dramatically enhances the prospect for the use of *Wolbachia* in a malaria control strategy, but many questions still remain. What are the effects of *Wolbachia* on the other four species of *Plasmodium* parasites that infect humans? How relevant are transient infection models? Do some strains of *Wolbachia* enhance pathogens in a field context? What are the long-term evolutionary consequences of novel *Wolbachia*-host associations on *Plasmodium* development within the insect host? What are the mechanisms behind pathogen interference and enhancement of *Wolbachia* on *Plasmodium* parasites, and are the mechanisms of enhancement seen in rodent and avian model systems relevant to human malaria parasites? How influential are environmental variables on pathogen inhibition phenotypes? While many of these questions may be difficult to answer in the short term, assessing the relevance of transient infections would seem within the grasp of the scientific community. Although challenging, understanding the evolutionary consequences of novel *Wolbachia* associations on pathogen transmission and identifying the mechanisms behind *Wolbachia* modulation of *Plasmodium* is critical for developing effective control strategies and assessing their long-term feasibility. Insights from non-Anopheline systems where *Wolbachia* naturally infects the vector may be useful in this regard [16], [31], [39].

In conclusion, *Wolbachia*-based control of vector-borne pathogens is a promising novel strategy that has many advantages over other conventional and contemporary control methods. The development of a stable infection in *Anopheles* means the prospect of *Wolbachia*-based control of malaria can now be entertained [25], but many important questions need to be resolved before this idea can become a reality. While the concerns raised here focus on *Plasmodium*, these issues are relevant for *Wolbachia* control of any vector-borne pathogen [18]; we suggest that transinfected mosquitoes intended for release into nature should be assessed for inhibition (or lack thereof) of all relevant pathogens circulating in the system.
